# Monocyte dysfunction in decompensated cirrhosis is mediated by the prostaglandin E2-EP4 pathway

**DOI:** 10.1016/j.jhepr.2021.100332

**Published:** 2021-08-04

**Authors:** Alexander A. Maini, Natalia Becares, Louise China, Thais H. Tittanegro, Amit Patel, Roel P.H. De Maeyer, Nekisa Zakeri, Tu Vinh Long, Lucy Ly, Derek W. Gilroy, Alastair O’Brien

**Affiliations:** 1 Institute of Liver and Digestive Health, University College London, London, UK; 2 Division of Medicine, University College London, London, UK; 3 Royal Free Hospital NHS Trust, London, UK; 4 Barts and The London School of Medicine and Dentistry, Queen Mary University of London, London, UK

**Keywords:** HLA-DR, TNF, IL6, LPS, Cyclo-oxygenase 1, Microsomal PGE synthase 1, Decompensated cirrhosis, ACLF, acute-on-chronic liver failure, AD, acute decompensation, CAID, cirrhosis-associated immune dysfunction, CM, classical monocytes, COX, cyclooxygenase, cPGES, cytosolic PGE synthase, CRP, C-reactive protein, DSS, downstream synthases, EIA, enzyme immune assay, FACS, polychromatic flow cytometric analysis, HVs, healthy volunteers, HLA DR, human leukocyte antigen – DR isotype, HPGD, 15-hydroxyprostaglandin dehydrogenase, LC-MS/MS, liquid chromatography-tandem mass spectrometry, LPS, lipopolysaccharide, MDMs, monocyte-derived macrophages, MFI, mean fluorescence intensity, mPGES1, microsomal PGE synthase 1, NASH, non-alcoholic steatohepatitis, OPD, patients with refractory ascites attending hospital outpatient department for day case paracentesis, PGE_2_, prostaglandin E_2_, qPCR, quantitative PCR, sCD14, soluble CD14, TIPS, transjugular intrahepatic portosystemic shunt insertion, TNFα, tumour necrosis factor alpha

## Abstract

**Background & Aims:**

Infection is a major problem in advanced liver disease secondary to monocyte dysfunction. Elevated prostaglandin (PG)E_2_ is a mediator of monocyte dysfunction in cirrhosis; thus, we examined PGE_2_ signalling in outpatients with ascites and in patients hospitalised with acute decompensation to identify potential therapeutic targets aimed at improving monocyte dysfunction.

**Methods:**

Using samples from 11 outpatients with ascites and 28 patients hospitalised with decompensated cirrhosis, we assayed plasma levels of PGE_2_ and lipopolysaccharide (LPS); performed quantitative real-time PCR on monocytes; and examined peripheral blood monocyte function. We performed western blotting and immunohistochemistry for PG biosynthetic machinery expression in liver tissue. Finally, we investigated the effect of PGE_2_ antagonists in whole blood using polychromatic flow cytometry and cytokine production.

**Results:**

We show that hepatic production of PGE_2_ via the cyclo-oxygenase 1–microsomal PGE synthase 1 pathway, and circulating monocytes contributes to increased plasma PGE_2_ in decompensated cirrhosis. Transjugular intrahepatic sampling did not reveal whether hepatic or monocytic production was larger. Blood monocyte numbers increased, whereas individual monocyte function decreased as patients progressed from outpatients with ascites to patients hospitalised with acute decompensation, as assessed by Human Leukocyte Antigen (HLA)–DR isotype expression and tumour necrosis factor alpha and IL6 production. PGE_2_ mediated this dysfunction via its EP_4_ receptor.

**Conclusions:**

PGE_2_ mediates monocyte dysfunction in decompensated cirrhosis via its EP_4_ receptor and dysfunction was worse in hospitalised patients compared with outpatients with ascites. Our study identifies a potential drug target and therapeutic opportunity in these outpatients with ascites to reverse this process to prevent infection and hospital admission.

**Lay summary:**

Patients with decompensated cirrhosis (jaundice, fluid build-up, confusion, and vomiting blood) have high infection rates that lead to high mortality rates. A white blood cell subset, monocytes, function poorly in these patients, which is a key factor underlying their sensitivity to infection. We show that monocyte dysfunction in decompensated cirrhosis is mediated by a lipid hormone in the blood, prostaglandin E_2_, which is present at elevated levels, via its EP_4_ pathway. This dysfunction worsens when patients are hospitalised with complications of cirrhosis compared with those in the outpatients setting, which supports the EP_4_ pathway as a potential therapeutic target for patients to prevent infection and hospitalisation.

## Highlights

• We demonstrate that monocyte dysfunction in patients with decompensated cirrhosis and refractory ascites is mediated by PGE_2_ through its EP_4_ receptor.
• Monocyte HLA-DR expression was also reduced in both cohorts of patients with decompensated cirrhosis, at least in part, by elevated circulating PGE_2_.
• Up to 55% of patients with ACLF have an infective precipitant.
• Improving monocyte function in patients with pre-hospital cirrhosis and ascites could prevent development of ACLF.
• PGE_2_-EP_4_ receptor antagonists might represent a treatment to improve monocyte dysfunction in outpatients with ascites.

## Graphical abstract

**Figure:**
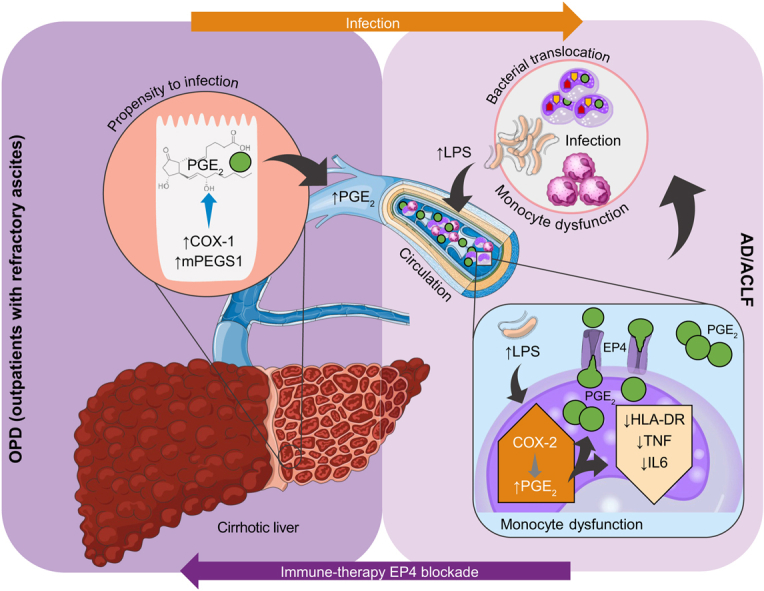

## Introduction

An estimated 2 million deaths worldwide are currently attributable to liver disease, a substantial increase from 676,000 (1.5%) in 1980.^1^^,^^2^ In patients with cirrhosis, bacterial infection or sepsis is a principal trigger for hospital admission leading to acute decompensation (AD) and acute-on-chronic-liver failure (ACLF), when accompanied by organ failure,^3^ drastically shortening life expectancy. Furthermore, although overall US inpatient cirrhosis mortality rates fell from 2002 to 2010, actual mortality risk from infection or sepsis increased.^4^

A dysregulated immune function underlies this increased propensity to infection, termed ‘cirrhosis-associated immune dysfunction’ (CAID).[5], [6], [7], [8], [9] We examined monocytes, because these key innate immune cells initiate and regulate the inflammatory response. Monocyte dysfunction has been defined as reduced monocyte human leukocyte antigen–DR isotype (HLA-DR) expression^10^ and reduced *ex vivo* lipopolysaccharide (LPS)-induced tumour necrosis factor alpha (TNFα) production,[11], [12], [13], [14], [15] and is associated with increased infection rates.[16], [17], [18] Several studies detail circulating monocyte dysfunction in advanced liver disease^11^^,^^19^ and persistently low expression of HLA-DR was associated with increased secondary infection and 28-day mortality.^20^

Elevated prostaglandin (PG)E_2_ is a mediator of monocyte/macrophage dysfunction in cirrhosis.^21^ Therefore, a therapeutic agent that reverses the effects of PGE_2_ could prevent the harmful effects of infection. NSAIDs reduce PGE_2_ production, but are contraindicated in decompensated cirrhosis because of nephrotoxicity.[22], [23], [24] Alternatively, albumin was found to antagonise the effects of PGE_2_.^15^ However, the Albumin To prevenT Infection in chronic liveR failurE (ATTIRE) trial showed no effect of targeted albumin infusions that achieved a serum albumin ≥30 g/L to hospitalised patients for development of infection, renal dysfunction ,or mortality compared with standard care.^25^ By contrast, the ANSWER trial (investigating albumin for the treatment of ascites in patients with hepatic cirrhosis) showed weekly albumin infusions to outpatients with ascites reduced the incidence of infection and improved survival.^26^ The differences between the ATTIRE and ANSWER trial outcomes suggest that interventions that combat the effects of PGE_2_ would be more effective if given to prehospital patients. Therefore, we compared patients hospitalised with decompensated cirrhosis (including both AD and ACLF) to those with ascites refractory to medical management attending the outpatient department for paracentesis (OPD) to identify a potential therapeutic target to improve monocyte function and prevent infection and hospital admission.

## Patients and methods

### Ethical approval

NHS Research Ethics Committee ethical approval was given for ‘An investigation of suppression of cirrhosis-mediated immune suppression by prostaglandin receptor antagonism’ (IRAS 170839, REC 15/LO/0800) with A.O’B. as the principal investigator and University College London Hospitals (UCLH) as the joint research office sponsor. All procedures were in accordance with the Helsinki Declaration of 1975, revised 1983. Patient and healthy volunteer recruitment was under this approval unless stated, with further approval from Health Research Authority in 2016. Our Patient and Public Involvement group advised on patient information sheets and consent forms.

### Inclusion criteria

The study included all patients attending day-case outpatient setting with ascites refractory to medical therapy for paracentesis (OPDs) or hospitalised with complications of decompensated liver cirrhosis (including AD and/or ACLF) aged over 18 years with predicted hospital length of stay greater than 5 days. Informed consent was obtained from all participants.

### Exclusion criteria

The exclusion criteria were: HIV infection; advanced hepatocellular carcinoma; patients not expected to survive >48 h; and pregnancy. Nonsmoking healthy volunteers (HVs) aged 18–50 provided samples for analyses.

### Patient demographics

Patients hospitalised with AD/ACLF were recruited from UCLH and OPDs from the day-case unit at The Royal Free Hospital, using standard diagnostic criteria (Table 1). In total, 11 OPDs were recruited at day-case paracentesis, none had infection at sampling, which was performed before albumin infusion and paracentesis, and none were taking antibiotics for treatment or prophylaxis. Overall, 28 patients with AD/ACLF were recruited up to day 9 following hospitalisation (Table 1). It was not possible to perform all experimental analyses on every patient and absolute numbers of samples used are detailed in the figure legends. Plasma samples were analysed from 17 patients undergoing transjugular intrahepatic portosystemic shunt insertion (TIPS) (Table 2). Hepatic venous sampling was prepuncture through the liver, whereas portal venous blood sampling was after catheter entry to the portal vein, ∼1 min after shunt insertion. Ethical approval from UCL-Royal Free Hospital biobank ref: 16/WA/0289.

**Table 1:** **Details of outpatients with refractory ascites and acute decompensation/acute-on-chronic liver failure**.

Characteristic	Outpatients with refractory ascites (n)	Patients with acute decompensation/acute-on-chronic liver failure (n)
Number	11	28
Age	60 (55–66)	56.5 (53–68)
Sex	Male (8)	Male (20)
Cirrhosis aetiology	Alcohol (7), NASH (2), autoimmune (1), drug (1)	Alcohol (24), NASH (2), HCV (1), primary biliary cholangitis (1)
Reason for admission (can be >1)	Paracentesis (11)	Infection (11), hepatic encephalopathy (9), gastrointestinal bleed (6), ascites (13), alcohol withdrawal (2), alcoholic hepatitis (1)
Location	Day-case unit	Ward (24), ICU (4)
Antibiotics	0	14
Model for end-stage liver disease score	15 (13–16)	17 (10–20)
Acute-on-Chronic Liver Failure score	n/a	Grade 0: n = 15
		Grade 1: n = 6
		Grade 2: n = 6
		Grade 3: n = 1
Death during admission	n.a.	9
Type 2 diabetes mellitus	3	7

NASH, non-alcoholic steatohepatitis.

**Table 2:** **Details of transjugular intrahepatic portosystemic shunt patients**.

Age	Sex	Aetiology of cirrhosis	TIPS indication
48	F	NASH	Ascites
50	M	Alcohol	Ascites
64	F	Primary sclerosing cholangitis	Variceal bleed
76	M	Alcohol/haemochromatosis	Ascites
50	F	Alcohol	Ascites
59	F	Alcohol	Ascites
58	M	HBV and HDV co-infection	Ascites
60	M	Primary biliary cholangitis	Hepatic hydrothorax
54	M	Cryptogenic	Ascites
69	F	Autoimmune hepatitis	Ascites
61	F	NASH	Variceal bleed
50	M	Alcohol	Variceal bleed
53	M	Alcohol	Ascites
60	F	Autoimmune hepatitis	Ascites
62	M	HCV	Ascites
61	F	Alcohol	Ascites
74	M	Alcohol	Ascites

NASH, non-alcoholic steatohepatitis; TIPS, transjugular intrahepatic portosystemic shunt.

### Polychromatic Flow Cytometric Analysis

Polychromatic flow cytometric analysis (FACS), including intracellular staining, defining the lineages of blood cells, was performed using a LSR Fortessa flow cytometer. See the supplementary material for methods, Supplementary Table S2 for antibody details, and Supplementary Figs S1 and S2 for the gating strategy used (lineage/activation panels).

### Quantitative real-time PCR

Quantitative real-time PCR (qPCR) of purified (bulk) monocytes was used to assess the expression of enzymes involved in the synthesis and catalysis of PGE_2_ [PLA2G4A, PTGS1, PTGS2, PTGES1, PTGES2, PTGES3, and 15-hydroxyprostaglandin dehydrogenase (HPGD)] and mRNA for the PGE_2_ membrane-bound receptors EP1–4 (*PTGER1*, *PTGER2*, *PTGER3*, and *PTGER4*). mRNA was extracted from samples using the RNeasy Mini Kit according to the manufacturer’s instructions and cDNA was synthesised using the High-Capacity cDNA Reverse Transcription Kit as per the manufacturer’s instructions.

### Immunohistochemistry

Patients with decompensated cirrhosis infrequently undergo liver biopsy; therefore, we examined cirrhotic liver explants from patients undergoing liver transplants at the Royal Free Hospital and specimens following resection of benign adenomas as healthy controls. For the details of the 10 liver explants examined, see the supplementary methods.

### Plasma PGE_2_ liquid chromatography-tandem mass spectrometry

Plasma samples were analysed by liquid chromatography-tandem mass spectrometry (LC-MS/MS) following protocols published previously.^27^ PGE_2_ and d_4_-PGE_2_ standards for LC-MS/MS analysis were purchased from Cayman Chemicals. Linear calibration curves were obtained for each lipid mediator, with r^2^ values of 0.98–0.99.

### Statistical analysis

The data were analysed using Graph Pad Prism 7.0. Unless stated, data are presented as mean ± SD. Where appropriate the following statistical tests were performed: (a) 1-way ANOVA and multiple comparisons using Browne–Forsythe, Tukey and Welch’s tests when 3 or more groups of values were compared; (b) Two-tailed (unpaired) *t* tests ± Welsh correction were performed when comparing 2 independent groups of values with normal distribution; (c) For the whole-blood experiments, data were analysed with 2-way ANOVA with multiple comparisons with Dunnett’s correction.

## Results

### Circulating PGE_2_ is elevated in patients hospitalised with decompensated liver cirrhosis compared with outpatients

**Fig. 1:**
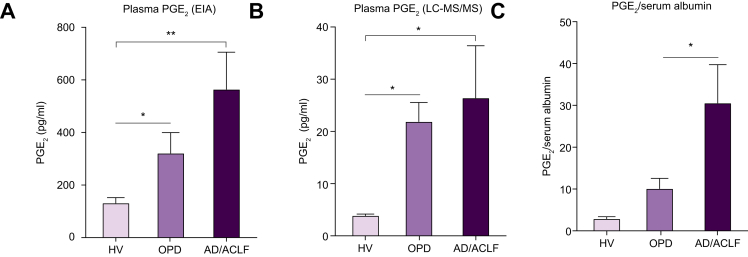
Plasma PGE_2_ concentration in decompensated cirrhosis. Plasma PGE_2_ concentrations in HVs, OPDs, and AD/ACLF using (A) EIA, results expressed as pg/ml from n = 12, 11, and 26 for HV, OPD, and AD/ACLF, respectively and (B) LC-MS/MS results expressed as pg/ml from n = 4 HVs, n = 9 OPDs, and n = 12 AD/ACLF. (C) Plasma PGE_2_ concentrations expressed according to corresponding serum albumin value in OPDs and patients with AD/ACLF. Data expressed as mean ± SEM, with 1-way ANOVA and multiple comparisons using Browne–Forsythe and Welch’s tests for patient comparisons with HVs. ∗*p* <0.05, ∗∗*p* <0.01. ACLF, acute-on-chronic liver failure; AD, acute decompensation; EIA, enzyme immune assay; HVs, healthy volunteers; LC-MS/MS, liquid chromatography-tandem mass spectrometry; OPD, patients with refractory ascites attending hospital outpatient department for day-case paracentesis; PGE_2_, prostaglandin E_2_.

Plasma PGE_2_ concentrations, based on LC-MS/MS and enzyme immune assay (EIA), were significantly elevated in patients with decompensated cirrhosis compared with HVs. Values were highest in patients hospitalised with AD/ACLF, whereas OPDs demonstrated intermediate levels (Fig. 1A,B). Most patients had alcohol-induced cirrhosis (Table 1). Although EIA measurements of PGE_2_ were consistently much higher than those from LC-MS/MS, these data demonstrated EIA reproducibly produced qualitative differences between sample groups consistent with data from LC-MS/MS analysis, highlighting EIA as a robust practical alternative to LC-MS/MS. Therefore, we used EIA for further analyses. The differences in PGE_2_ between liver disease cohorts did not reach significance using either technique. However, we showed previously that circulating albumin binds to, and inactivates, PGE_2_.^11^ Therefore, the lower serum albumin concentrations in patients with AD/ACLF compared with OPDs (*p* <0.05, Supplementary Table S1A) indicated that less of the total PGE_2_ measured was albumin bound and, consequently, more unbound, bioavailable PGE_2_ was present in these patients. To demonstrate this, the PGE_2_ levels (EIA) of patients were expressed according to their corresponding serum albumin values, which demonstrated significant differences between HVs, OPDs and AD/ACLF (*p* <0.01, Fig. 1C).

### Elevated circulating PGE_2_ in patients with decompensated liver cirrhosis is produced by both the cirrhotic liver and peripheral blood monocytes

**Fig. 2:**
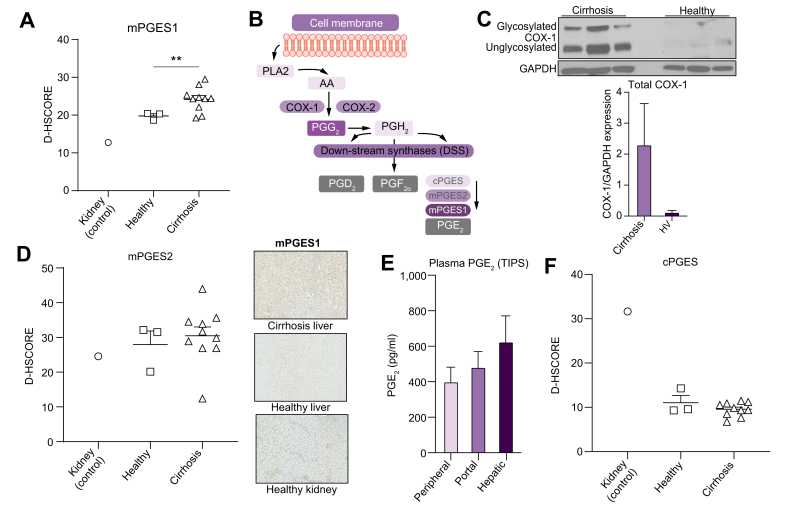
Hepatic production of PGE_2_ in cirrhosis. (A) Plasma PGE_2_ from portal, hepatic, and peripheral blood from 17 patients undergoing TIPS. (B) Representative pathway of PGE_2_ synthase. (C) Western blot protein expression of COX-1 within cirrhotic and healthy livers with results normalised to expression of the reference protein GAPDH (n = 3 per group). Immunohistochemical analyses in healthy and cirrhotic livers and healthy kidneys (with representative photos in Fig. S4) of (D) mPGES1 synthase expression, (E) mPGES2, and (F) cPGES. Digital H-Scores presented for liver sections stained with each antibody for 3 groups (kidney n = 1, healthy liver n = 3, cirrhotic liver n = 10). COX, cyclooxygenase; cPGES, cytosolic PGE synthase; mPGES, microsomal PGE synthase; PGE_2_, prostaglandin E_2_; TIPS, transjugular intrahepatic portosystemic shunt insertion.

Samples from patients undergoing TIPS (Table 2) demonstrated levels of PGE_2_ that were higher in hepatic venous blood compared with peripheral (average of 1.7-fold) and portal venous blood (1.44-fold), although the differences did not achieve statistical significance and firm conclusions could not be drawn as to the major source of PGE_2_ production (Fig. 2A).

Prostaglandins are formed by phospholipase A_2_ (PLA_2_) releasing arachidonic acid from the plasma membrane, which is metabolised by constitutive cyclooxygenase 1 (COX-1) or inducible COX-2 to PGH_2_ via PGG_2_. PGH_2_ acts as substrate for downstream synthases (DSS), PGE_2_, PGD_2_, prostacyclin, and PGF_2_α. Microsomal PGE synthase 1 (mPGES1), mPGES2, and constitutively expressed cytosolic (c)PGES/p23 convert PGH_2_ to PGE_2_; mPGES1 is the main inducible synthase following proinflammatory stimuli.^28^

We compared cirrhotic liver explants following transplantation with nondiseased livers following resection for benign adenomas as healthy controls. Western blot revealed upregulated COX-1 protein within cirrhotic liver tissue (Fig. 2B), with COX-2 undetectable in cirrhotic and healthy liver tissue (data not shown). With limited liver tissue availability, we determined the expression of PGE_2_ DSS using immunohistochemistry and kidney as a positive control with a known presence of PGE_2_ DSS.^29^ Cirrhotic livers showed increased mPGES1 compared with healthy livers, with a digital H-Score of 24.17 *vs*. 19.79 (*p* <0.01, Fig. 2C). There were no differences in mPGES2 or cPGES (Fig. 2D,E). Thus, these data support the cirrhotic liver as a source of increased plasma PGE_2_ in decompensated cirrhosis via upregulation of COX-1/mPGES1.

**Fig. 3:**
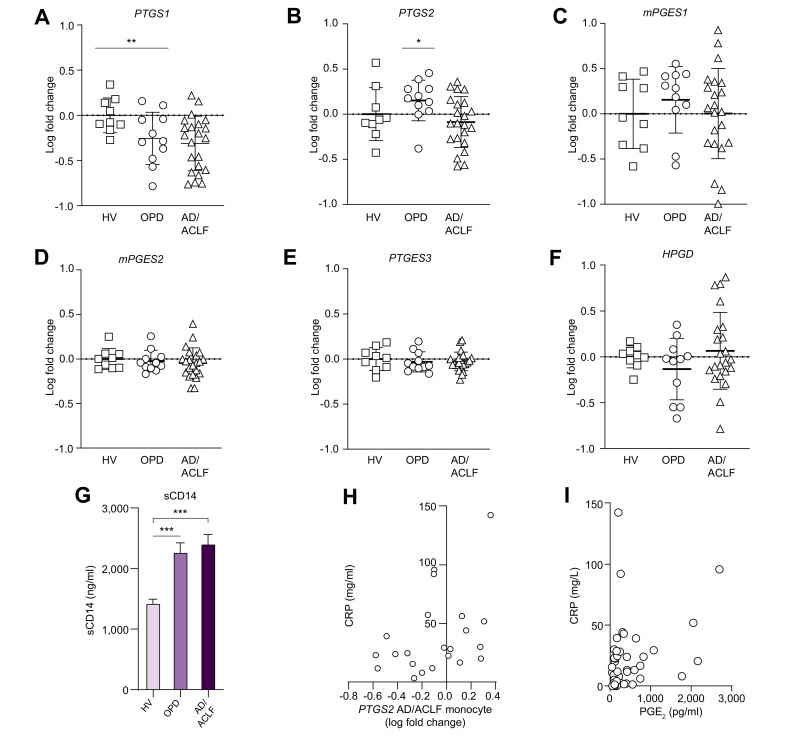
Monocyte gene expression for PGE_2_ synthases and circulating endotoxin levels in decompensated cirrhosis. mRNA levels of (A) *PTGS1* (COX-1), (B) *PTGS2* (COX-2), (C) *mPGES1*, (D) *mPGES2*_,_ (E) *PTGES3* (cPGES) synthase enzymes, and (F) *HPGD* in purified primary human monocytes from n = 9, 11, and 22 for HVs, OPDs, and AD/ACLF, respectively. (G) Plasma soluble CD14 (sCD14) in n = 12, 11, and 26 for HVs, OPDs, and AD/ACLF, respectively. (H) Correlation between plasma CRP and *PTGS2* (COX-2). (I) Correlation between plasma CRP and plasma PGE_2_ concentrations using Pearson’s correlation coefficient followed by Bonferroni correction. Data expressed as mean ± SEM or individual data plots, unpaired *t*-test ± Welsh correction for patient comparisons with HVs. qPCR results normalised to the reference gene *SNRPD3* and data expressed as fold change relative to healthy levels with 1-way ANOVA and multiple comparisons performed using Browne–Forsythe and Welch’s tests. ∗*p* <0.05, ∗∗*p* <0.01. ACLF, acute-on-chronic liver failure; AD, acute decompensation; CRP, C-reactive protein; DSS, downstream synthases; HVs, healthy volunteers; HPGD, 15-hydroxyprostaglandin dehydrogenase; OPD, patients with refractory ascites attending hospital outpatient department for day-case paracentesis; PGE_2_, prostaglandin E_2_; qPCR, quantitative PCR; sCD14, soluble CD14.

Peripheral blood qPCR revealed a significant reduction in blood monocyte *PTGS1* (COX-1) gene expression in AD/ACLF compared with healthy controls (*p* <0.01) with OPDs demonstrating intermediate expression (Fig. 3A). *PTGS2* (COX-2) gene expression was reduced when OPDs were compared with AD/ACLF (*p* <0.05), with *mPGES1* expression unchanged across healthy and cirrhosis cohorts (Fig. 3B,C). There were no differences in other PGE_2_ synthesis or catabolism enzymes, mPGES2, PTGES3, or HPGD (Fig. 3D–F). We observed increased plasma soluble CD14 (sCD14), a co-receptor for LPS (endotoxin) released by monocytes upon activation in patients with decompensated cirrhosis compared with HVs (Fig. 3G). Given that LPS in plasma will trigger PGE_2_ production in the presence of monocyte COX enzymes,^10^ these data suggest that circulating monocytes contribute to PGE_2_ production via LPS-COX stimulation in decompensated cirrhosis, although COX enzyme expression was lower than in healthy controls.

### Increased systemic inflammation in patients with AD/ACLF might trigger increased COX-2 production of PGE_2_ in peripheral blood monocytes

COX-2 expression on monocytes was highly variable in patients with AD/ACLF compared with OPDs (Fig. 3B). Investigating this further, we found COX-2 expression positively correlated with plasma C-reactive protein (CRP) in patients with AD/ACLF (CRP, r^2^ = 0.4, *p* = 0.03, Fig. 3H), suggesting that those with increased systemic inflammation had greater monocyte COX-2 expression. Consistent with these analyses, plasma CRP also correlated positively with plasma PGE_2_ in samples tested (r^2^ = 0.33, *p* = 0.02, Fig. 3I). These data suggest that monocyte contribution to PGE_2_ production is highest in patients with AD/ACLF with the greatest systemic inflammation.

**Fig. 4:**
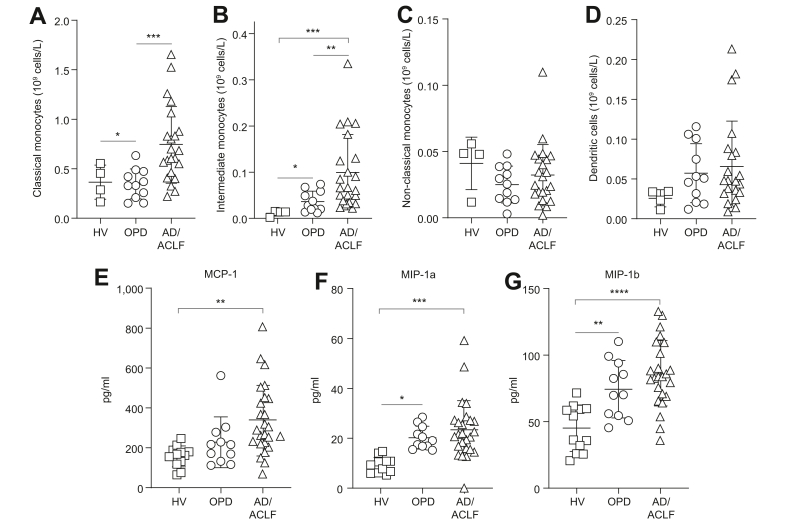
Monocytes subsets and PGE_2_-mediated chemokine levels in decompensated cirrhosis. (A–D) Monocyte cell counts according to flow cytometry analysis, with classical (A), intermediate (B), nonclassical (C), and dendritic cells (D), n = 4, 11, and 23 for HVs, OPDs, and AD/ACLF, respectively. The PGE_2_-mediated plasma chemo/cytokines MCP-1 (E), MIP-1a (F), and MIP-1b (G); n = 9, 11, and 25 for HVs, OPDs, and AD/ACLF, respectively. Data expressed as mean ± SD analysed with 1-way ANOVA with Tukey multiple comparisons test (all groups compared). ∗*p* <0.05, ∗∗∗*p* <0.001, ∗∗∗∗*p* <0.0001. ACLF, acute-on-chronic liver failure; AD, acute decompensation; HVs, healthy volunteers; OPD, patients with refractory ascites attending hospital outpatient department for day-case paracentesis; PGE_2_, prostaglandin E_2_.

Consistent with elevated circulating LPS, patients with AD/ACLF had an expansion of total monocyte numbers, specifically relating to classical (*p* = 0.003) and intermediate monocytes (*p* = 0.02), with an intermediate phenotype in OPDs, and no changes in nonclassical monocytes and dendritic cells across all cohorts (Fig. 4A–D).

Finally, there were significant increases in PGE_2_-mediated plasma chemokines, MCP-1, MIP-1a, and MIP-1b, in patients with AD/ACLF, with an intermediate phenotype in OPDs (Fig. 4E–G).

### Monocyte dysfunction is more severe in patients hospitalised with decompensated cirrhosis compared with outpatients

Monocyte dysfunction was assessed by examining HLA-DR expression and whole-blood LPS-induced TNFα production. We included IL6 production in line with its crucial role in patients with AD/ACLF.^30^^,^^31^

**Fig. 5:**
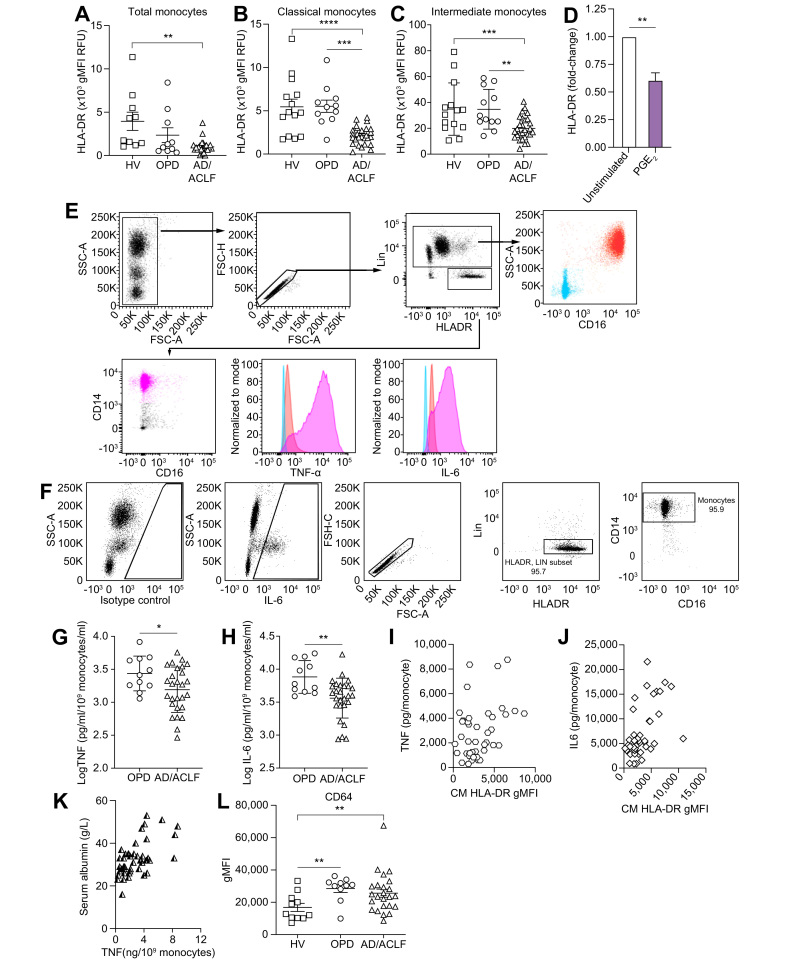
Monocyte dysfunction in decompensated cirrhosis. Geometric MFI of HLA-DR cell surface expression on (A) all monocytic cells, (B) classical, and (C) intermediate monocytes in n = 11, 10, and 24 for HVs, OPDs, and AD/ACLF, respectively. (D) Monocyte HLA-DR expression by flow cytometry following incubation of HV whole blood ±1 ng/ml of PGE_2_ for 72 h (n = 3). TNFα (E) and IL6 (F) expression in HV monocytes following LPS stimulation. (G) TNFα and (H) IL6 at 4 h in n = 21, 11, and 28 for HVs, OPDs, and AD/ACLF, respectively, with the log [TNFα/IL6 normalised to monocyte number] displayed. Correlation between baseline MFI HLA-DR expression on CM cell population with subsequent whole blood (I) TNFα and (J) IL6 production following LPS stimulation (n = 9, 11, and 28 for HVs, OPDs, and AD/ACLF, respectively). (K) Correlation between LPS-stimulated monocyte TNFα production and serum albumin in decompensated cirrhosis. (L) MFI of CD64 cell surface expression on all monocytic cells in n = 11, 10, and 24 for HVs, OPDs, and AD/ACLF, respectively. Individual data points or mean ± SEM shown with 1-way ANOVA with Tukey multiple comparisons test (all groups compared) or unpaired *t*-test when 2 groups. ∗*p* <0.05, ∗∗*p* <0.01, ∗∗∗*p* <0.001, ∗∗∗∗*p* <0.0001. ACLF, acute-on-chronic liver failure; AD, acute decompensation; CM, classical monocytes; HLA-DR, human leukocyte antigen – DR isotype; HVs, healthy volunteers; LPS, lipopolysaccharide; MFI, mean fluorescence intensity; PGE_2_, prostaglandin E_2_; OPD, patients with refractory ascites attending hospital outpatient department for day-case paracentesis; TNFα, tumour necrosis factor alpha.

There was a reduction in HLA-DR surface expression (*p* = 0.03, Fig. 5A), mostly in classical and intermediate monocytes in patients with AD/ACLF, but not in OPDs (Fig. 5B,C). Linking this to the increased bioavailable circulating PGE_2_ in AD/ACLF compared with OPD, we found that HLA-DR expression on monocytes fell significantly following 72 h incubation with 1 ng/ml PGE_2_ compared with untreated controls (Fig. 5D). Patients with sepsis and lower HLA-DR^+^ monocytes have more secondary infections and worse outcomes,^31^ supporting the potential functional relevance of differences in HLA-DR expression between OPDs and patients with AD/ACLF .

Monocytes, especially classical and intermediate, make TNFα and IL6 following whole-blood *ex vivo* LPS stimulation (Fig. 5E,F). Expressing LPS-stimulated TNFα and IL6 per blood monocyte number, synthesis of both cytokines fell significantly when comparing OPDs and patients hospitalised with AD/ACLF (Fig. 5G,H).

There was a close correlation between baseline HLA-DR expression on classical monocytes (the largest population) and TNFα (Fig. 5I; r = 0.3847, *p* = 0.0069) and IL-6 (Fig. 5J; r^2^ = 0.3, *p* = 0.0002) following LPS whole-blood stimulation. Furthermore, serum albumin levels in patients strongly correlated with monocyte LPS-stimulated TNFα release (Fig. 5K; r = 0.593, *p* <0.0001), supporting a link between PGE_2_ regulation by albumin and monocyte dysfunction. Not all monocyte expression markers were downregulated, with increased monocyte CD64 expression, similar to murine acute liver failure and sepsis (Fig. 5L).^32^^,^^33^

Collectively, our data demonstrate that the progression of patients from outpatients with ascites to patients hospitalised with AD/ACLF is accompanied by an increase in bioavailable PGE_2_, reduced monocyte HLA-DR expression, and reduced monocyte TNFα/IL6 production.

### PGE_2_ induces monocyte dysfunction in decompensated cirrhosis via its EP_4_ receptor

**Fig. 6:**
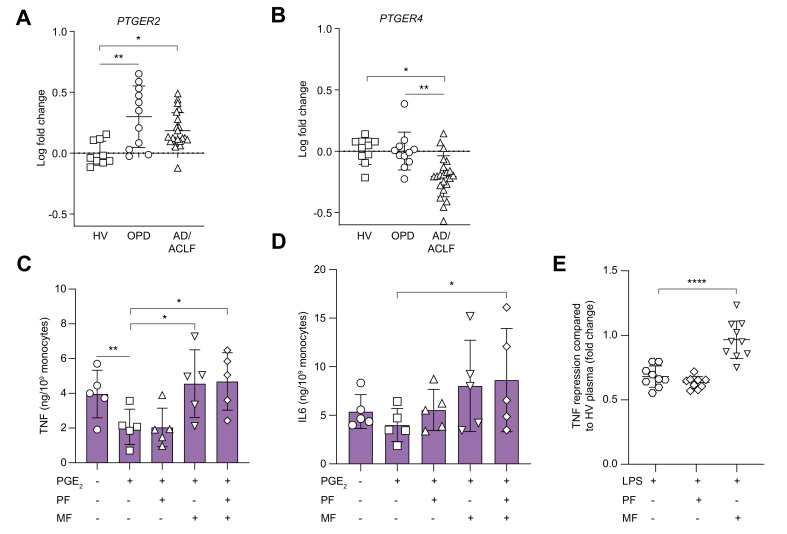
PGE_2_ induces monocyte dysfunction in decompensated cirrhosis through its EP_4_ receptor. (A) Monocyte PGE_2_-EP_2_ (*PTGER2)* and (B) PGE_2_-EP_4_ (*PTGER4)* receptor expression by qPCR. qPCR of purified isolated monocytes with log [fold change] plotted with mean of HV values set at 0 (*i.e.* fold change of 1) (n = 9, 11, and 22 for HV, OPDs, and AD/ACLF, respectively). Effect of 1 ng/ml PGE_2_ on LPS-induced TNFα (C) and IL6 (D) production/monocyte in AD/ACLF whole blood in presence/absence of pretreatment with the EP_2_ antagonist, PF-04418948 (1 μM) or EP_4_ receptor antagonist MF498 (10 μM) or both (n = 5 per group). (E) Monocyte-derived macrophages from healthy donors treated with plasma from OPDs and LPS activation quantified by TNFα production in presence or absence of PF-04418948 (1 μM) or MF498 (1 μM) (n = 9 to 10 per group) with results expressed as TNFα repression (fold-change) to MDMs treated with HV plasma. For whole-blood experiments, data were analysed with 2-way ANOVA with multiple comparisons with Dunnett’s correction (LPS + PGE_2_ as control for each cohort). For MDM experiments, data were analysed with 1-way ANOVA with Brown–Forsythe and Welch tests for multiple comparisons. ∗*p* <0.05, ∗∗*p* <0.01, ∗∗∗*p* <0.001, ∗∗∗∗*p* <0.0001. ACLF, acute-on-chronic liver failure; AD, acute decompensation; HV, healthy volunteer; LPS, lipopolysaccharide; MDMs, monocyte-derived macrophages; OPD, patients with refractory ascites attending hospital outpatient department for day-case paracentesis; PGE_2_, prostaglandin E_2_; qPCR, quantitative PCR; TNFα, tumour necrosis factor alpha.

qPCR of blood monocytes demonstrated upregulation of the EP_2_ receptor (*PTGER2*) mRNA in both OPDs and patients hospitalised with AD/ACLF compared with HVs (Fig. 6A). By contrast, monocyte EP_4_ receptor (*PTGER4*) mRNA was decreased in AD/ACLF, with no change in OPDs compared with HVs (Fig. 6B); EP_1_ (*PTGER1*) and EP_3_ (*PTGER3*) were very low or undetectable (data not shown).

To investigate whether EP_2_ and/or EP_4_ receptors transduced the effects of PGE_2_, whole blood from patients with AD/ACLF was pre-incubated with either PF-04418948, an EP_2_ selective antagonist (IC_50_ = 16 nM with >2,000-fold selectivity over EP_1_, EP_3_, and EP _4_) or MF498, a selective EP_4_ receptor antagonist (K_i_ = 0.7 nM *vs*. a K_i_ >1 μM for other EP receptors). Thereafter, cells were treated with PGE_2_ before LPS stimulation. Only pre-incubation using the EP_4_ receptor antagonist (MF498) completely reversed PGE_2_-suppressive effects on monocyte TNFα secretion from patients with AD/ACLF, with no additional effect when EP_2/4_ inhibitors were combined (Fig. 6C). PGE_2_-mediated suppression of IL6 exhibited a similar profile, although EP_4_ inhibition alone did not significantly reverse this, whereas the combination of EP_2/4_ inhibitors did (*p* <0.05, n = 5 per group) (Fig. 6D).

In the absence of sufficient whole blood from OPDs for these experiments, monocyte-derived macrophages (MDMs) from healthy donors were treated with plasma from OPDs or healthy controls, as previously described.^11^ Consistent with whole-blood studies, when LPS-induced TNFα production by MDMs in the presence of OPD plasma was compared with HVs, there was a significant reduction in TNFα production (*p* <0.05, n = 6). This was fully reversed by the EP_4_ receptor antagonist, MF498 (*p* <0.0001, n = 10) with no effect using the EP_2_ antagonist, PF-04418948 (Fig. 6E).

These findings demonstrate that elevated circulating PGE_2_ causes monocyte dysfunction in decompensated cirrhosis via its EP_4_ receptor, despite the relative increased expression of EP_2_ over EP_4_.

## Discussion

This study demonstrates that monocyte dysfunction in both patients hospitalised with AD/ACLF and OPDs is mediated by PGE_2_ through its EP_4_ receptor, with patients with AD/ACLF exhibiting a more severe phenotype. We focused principally on monocyte TNFα production because of its crucial role in the inflammatory cascade and data linking reduced monocyte TNFα release to survival following sepsis,[34], [35], [36], [37], [38] although a similar pattern was observed with IL6. Monocyte HLA-DR expression was also reduced in decompensated cirrhosis mediated, at least in part, by elevated circulating PGE_2_. We believe that our crucial observation was the change in monocyte dysfunction between outpatients and those in hospital, and this creates a potential therapeutic opportunity. Up to 55% of patients with ACLF have an infective precipitant^3^; therefore, improving monocyte function in patients with prehospital cirrhosis with ascites could prevent the development of this condition. Furthermore, a substantial gain could be achieved by keeping these prehospital patients stable, to allow time to address alcohol dependency, or undergo treatment for viral hepatitis or with emerging non-alcoholic steatohepatitis (NASH) therapies. Thus, PGE_2_-EP_4_ receptor antagonists could represent a treatment to improve monocyte dysfunction in outpatients with ascites.

The more severe PGE_2_-mediated monocyte dysfunction in patients hospitalised with AD/ACLF compared with OPDs could partly explain the lack of effect of albumin infusions in the prevention of infection in the ATTIRE trial, compared with the reduction in infections in the ANSWER trial.^25^^,^^26^ Infusing albumin might be able to overcome the less severe PGE_2_-mediated monocyte dysfunction in OPDs, but not in patients hospitalised with AD/ACLF.

The cirrhotic liver showed upregulated COX-1 and mPGES1. The latter regulates macrophage polarisation and liver protection and repair through EP_4_ signalling during hepatic ischemia-reperfusion injury in mice and is increased in human NASH livers.^39^ During early stages of liver injury, it appears that activation within the liver of the mPGES1 antifibrotic pathway is protective, but once decompensated cirrhosis occurs, the consequent chronic PGE_2_ production leads to monocyte dysfunction. However, the therapeutic potential of mPGES1 inhibitors to reverse PGE_2_-mediated monocyte dysfunction in decompensated cirrhosis appears limited, with no drug targeting this pathway progressing beyond safety studies.

We detected elevated circulating LPS secondary to gut bacterial translocation. This triggers PGE_2_ production via monocyte COX enzymes, even though COX levels were lower or equal to those in HVs, and this might also represent a significant source of PGE_2_.^10^ Targeting gut bacterial translocation using antibiotic prophylaxis to reduce LPS-induced monocyte PGE_2_ production could improve monocyte dysfunction. Indeed, persistent gut translocation of bacterial products causes immune exhaustion^9^; however, concerns over antimicrobial resistance limit the use of this approach.^40^ Non-antibiotic therapies to reduce gut bacterial translocation might also improve PGE_2_-induced monocyte dysfunction.^41^

We previously showed increased COX-2 in peripheral blood mononuclear cells in a small cohort of inpatients with AD/ACLF.^21^ Here, with a greater sample size, we show a disease-dependent reduction in blood monocyte COX-1 with no overall change in COX-2 between healthy and decompensated cirrhosis, although COX-2 expression fell between outpatients with ascites and those with AD/ACLF. There was significant heterogeneity in COX-2 expression in AD/ACLF and circulating PGE_2_ levels correlated positively with serum CRP, supporting increased COX-2 expression in patients with AD/ACLF with the greatest systemic inflammation or infection. The presence of systemic inflammation in patients sampled could explain the differences between our current and previous analyses.

Our study has limitations. Patients studied predominantly had alcohol-induced liver cirrhosis and analyses might differ for other aetiologies. We could not perform *in vivo* pharmacology studies on patients and these were not attempted in rodents, as rodent models of acute decompensated cirrhosis are not considered representative of the human phenotype.^42^ We only examined peripheral blood monocytes, and other immune cells are also dysfunctional in decompensated cirrhosis; for example, lymphoid cells (including T, B and natural killer cells) are decreased^43^^,^^44^ and neutrophils might have defective production of antimicrobial superoxide anions.^45^ Furthermore, we focussed solely on PGE_2_, and there are other potential targets to reverse monocyte–macrophage dysfunction in liver disease, such as Mer tyrosine kinase.^46^ Finally, our study was designed to detect differences between outpatients with ascites and patients with hospitalised decompensated cirrhosis; therefore, there were insufficient numbers to investigate whether monocyte function worsened according to ACLF grade, decompensated cirrhosis status,^47^ presence or absence of infection, and antibiotic prescription. It appears likely that these will all be significant confounders in hospitalised patients.

To conclude, we provide evidence entirely from human experimentation that demonstrates PGE_2_, acting via its EP_4_ receptor, downregulates monocyte TNFα and IL6 production in decompensated cirrhosis and reduces monocyte HLA-DR expression. Crucially, we observed worsening monocyte dysfunction in patients attending for day-case paracentesis compared with those hospitalised with AD/ACLF. Drug development based on our findings could lead to a proactive approach to improve monocyte dysfunction in outpatients with ascites to prevent infection and subsequent hospitalisation.

## Financial support

The work was supported mainly by the 10.13039/501100000265Medical Research Council UK (grant number MR/M005291/1). Support was also provided by the Health Innovation Challenge fund awarded to A.O’B. (Wellcome Trust and Department of Health and Social Care; HICF reference HICF-R8-439, WT grant number WT102568). This is a report of EudraCT 2014-002300-24 and International Standard RCT Number: 14174793. Research Ethics Committee Number: 15/LO/0104 and the Rosetrees Trust (award no. JS15/M728). The funders of the study had no role in the study design, data collection, data analysis, data interpretation, writing of the report, or views expressed in this publication.

## Authors’ contributions

A.A.M. performed most of the laboratory work and analysis greatly assisted by N.B. L.C. and A.P. performed additional laboratory work. N.Z. consented patients and obtained the transjugular intrahepatic portosystemic samples. T.V.L. obtained liver samples for immunohistochemistry. L.L. performed the lipid metabolomic work**.** A.O’B. and D.W.G. designed the laboratory experimental studies and A.O’B., D.W.G., T.H.T., R.D.M.. and A.A.M. co-wrote the manuscript. All authors had access to study data and reviewed and approved the final manuscript.

## Conflicts of interests

None of the authors have any conflict of interests or disclosures relevant to this manuscript.

Please refer to the accompanying ICMJE disclosure forms for further details.

## Data Availability

The data that support the findings of this study are available from the authors, AAM and AOB, upon reasonable request.
