# Supplementary material for: Monocyte dysfunction in decompensated cirrhosis is mediated by the prostaglandin E2-EP4 pathway
Source: JHEP Rep. 2021 Aug 4;3(6):100332. doi: 10.1016/j.jhepr.2021.100332 (PMC8603213; doi:10.1016/j.jhepr.2021.100332)
Supplement: Multimedia component 1 [file mmc1.pdf]

Monocyte dysfunction in decompensated cirrhosis is mediated by the prostaglandin E2-EP4 pathway

Alexander A Maini, Natalia Becares, Louise China, Thais H Tittanegro, Amit Patel, Roel PH De Maeyer, Nekisa Zakeri, Tu Vinh Long, Derek W Gilroy, Alastair O'Brien

Table of contents

Supplementary methods..2

Supplementary figures...6

Supplementary tables...14

Supplementary references..19

Supplementary methods

Peripheral Whole Blood (WB) *ex vivo* stimulation with endotoxin (LPS): Venous WB was collected in lithium heparin containing tubes. 1ml WB was diluted 1:5 in RPMI (Gibco, Grand Island, NY) in 15ml polypropylene tubes (Fisher Scientific, Grand Island, NY) and stimulated with 1ng/ml of LPS (*Salmonella abortus equi* S-form [TLR*grade*], Enzo Life Sciences, Farmingdale, NY), within 30 minutes of draw, for 4 hours (37°C). After incubation, samples were centrifuged at 2000g for 10 min at 20°C. Supernatant was removed and frozen at -80°C and remaining pellet lysed with 2ml ACK lysis buffer (Lonza, Basel, Switzerland) before centrifuged at 500g for 5 minutes at 10 ^{o}C. The cell pellet was resuspended in buffer and kept on ice before processing. Supernatants were processed for TNF, IL6 and soluble CD14 using single-analyte enzyme linked immunosorbent assay (EIA). In some experiments PGE_2 1ng/ml was added to samples ± selective EP_2 antagonist PF-04418948 (1 µM) or selective EP_4 receptor antagonist MF498 (10 µM) or both in combination (Cayman Chemicals, MI, USA). All samples were run in triplicate and mean taken before interpolation from the standard curve.

Monocyte-derived macrophages (MDM) *ex-vivo* immune function assay: MDMs were obtained as published previously (21) and stimulated with 1 ng/ml of lipopolysaccharide (*Salmonella abortus equi* S-form [TLR*grade*], Enzo Life Sciences, Farmingdale, NY) in presence/absence of EP_2 antagonist PF-04418948 (1 µM) or EP_4 antagonist MF498 (1 µM). Results were expressed as repression of TNF produced per well by macrophages when OPD treated macrophages were compared with HV treated macrophages (fold-change) i.e. HV-treated macrophage mean value was 1. This was in order to account for differences in absolute TNF values on the 2 different days that the experiments were performed.

Quantitative Real-Time PCR: Three reference genes were chosen according to their stability of expression in immune cells from literature and according to the Minimum Information for Publication of Quantitative Real-Time PCR Experiments (MIQE) Guidelines. Primers were selected from the ThermoFisher Scientific Taqman Database according to a number of parameters to ensure optimal qPCR quality: exon spanning probes, short amplicon length (<120 base pairs) and inventory availability. Taqman Primers **(Table S3)** were assessed for efficiency by performing serial dilutions of cDNA template and assessing CT values at each point. Linear regression was performed on all of these lines to fold induction or fold change then logged to produce graphs with no change set at 0 (i.e. fold change of 1, no change). This ensured increased and decreased expression were expressed equally in graphic form. For example, a 10-fold induction becomes a value of 1 on a graph, and a 10-fold reduction (i.e. relative expression of 0.1) becomes -1. This approach averts biased interpretations.

Section Analysis and Scoring: Slides were scanned using the NanoZoomer Whole Slide Imaging System (Hamamatsu Photonic (UK) Ltd) at 20x before being analysed using ImageJ (FIJI version, FIJI contributors, NIH, USA). Three randomly selected areas of stained slide were taken at 10x zoom before colour deconvoluting into their three component colours. The diaminobenzidine (DAB) shows up in one of these images only. The median intensity of the signal for the whole image was taken. To provide a score, this was taken away from 255 (maximum intensity) and the scores from the three areas of the slide averaged to produce a mean. This score is the 'D-HSCORE' that gives a score in optical density units (OD). These scores provide an objective measure of staining (1-3).

Polychromatic Flow Cytometric Analysis (FACs): Isolated leukocyte cell populations were depleted of erythrocytes by ACK lysis buffer and re-suspended in buffer (FACS buffer: 5% FBS in PBS), counted, and aliquots of 0.2-1x10^6 cells/condition made in FACs tubes. Cells were incubated with Human Trustain FcX (Fc Receptor Blocking Solution, Biolegend UK) to prevent non-specific antibody binding. Cells were analysed using LSR Fortessa (BD Biosciences) with 4 lasers (Violet (405nm), Blue (488), Green (561nm), Red (640nm)) within 48 hours of staining. Gating for data acquisition used BD FACSDiva 8.0.1. Population Flow and cytometry data were analysed using FlowJo v10.0.7 (Tree Star, Inc.). Results are expressed as a percentage of single cells and/or geometric mean fluorescence intensity (MFI). Compensation controls were used for each fluorochrome to control for spectral overlap.

Monocytes isolation: Monocytes were isolated from EDTA-venous blood using negative selection RosetteSep Human Monocyte Enrichment Cocktail (STEMCELL Technologies). Fresh whole blood was incubated for 20 minutes with RosetteSep Human Monocyte Enrichment Cocktail (STEMCELL Technologies) followed by density gradient centrifugation (1200xg/10mins/20°C) with Ficoll-Paque (Sigma-Aldrich) in 50ml SepMate tubes (STEMCELL Technologies). Cells were counted and resuspended for live assays, lysed in RLT buffer (Qiagen) for RNA extraction and purification or frozen at -80°C as pellets.

Western Blotting: Frozen liver samples from cirrhotic liver explants as described above were also used. Cell pellets were lysed in RIPA buffer (Sigma Aldrich, MO, USA) supplemented with protease inhibitors. 30 µg of cell lysates were loaded onto a 4-15% Tris-Glycine gel (Bio-Rad, CA, USA), electrophoresed and transferred onto a PVDF membrane. Membranes were immunoblotted with the following primary

antibodies, rabbit polyclonal anti COX-1 (4841, Cell Signaling, MA, USA), rabbit polyclonal anti COX-2 (160126, Cayman) and mouse monoclonal anti-GAPDH (sc-32233, Santa Cruz Biotechnology, TX, USA. Anti-rabbit (sc-2301, Santa Cruz Biotechnology, TX, USA) or anti-mouse (NA931VS, GE Healthcare) horseradish peroxidase–tagged antibodies were used for secondary binding. Chemiluminescence (Clarity Western ECL, Bio-Rad, CA, USA) was used to visualise proteins.

Plasma PGE_2 Enzymeimmunoassay (EIA) measured PGE_2 plasma concentration using Amersham Prostaglandin E_2 Biotrak Enzymeimmunoassay System (GE Healthcare). As manufacturer instructions, samples were hydrolysed and total PGE_2 was measured (detection of both free and bound to albumin PGE_2). Unknown concentrations were determined via comparison to control and samples run in triplicate with mean taken.

Plasma chemokines were measured by electrochemiluminescence using *V-Plex* Chemokine Panel 1 kits, Meso Scale Discovery, Rockville, Maryland, USA.

Immunohistochemistry: The 10 liver explants were: alcoholic liver disease-3, viral hepatitis-3, primary sclerosing cholangitis-2, primary biliary cholangitis-1, NASH cirrhosis-1 and Wilson's disease-1. Approval - Royal Free Hospital Ethics Committee (07/Q0501/50). Immunohistochemistry staining for PGE_2 synthesis and catabolism enzymes was performed on liver explants and resections using Ventana Discovery XT instrument, Ventana DAB Map detection Kit (760-124) and counterstained with haemotoxylin **(see Fig. S4)**.

Supplementary figures

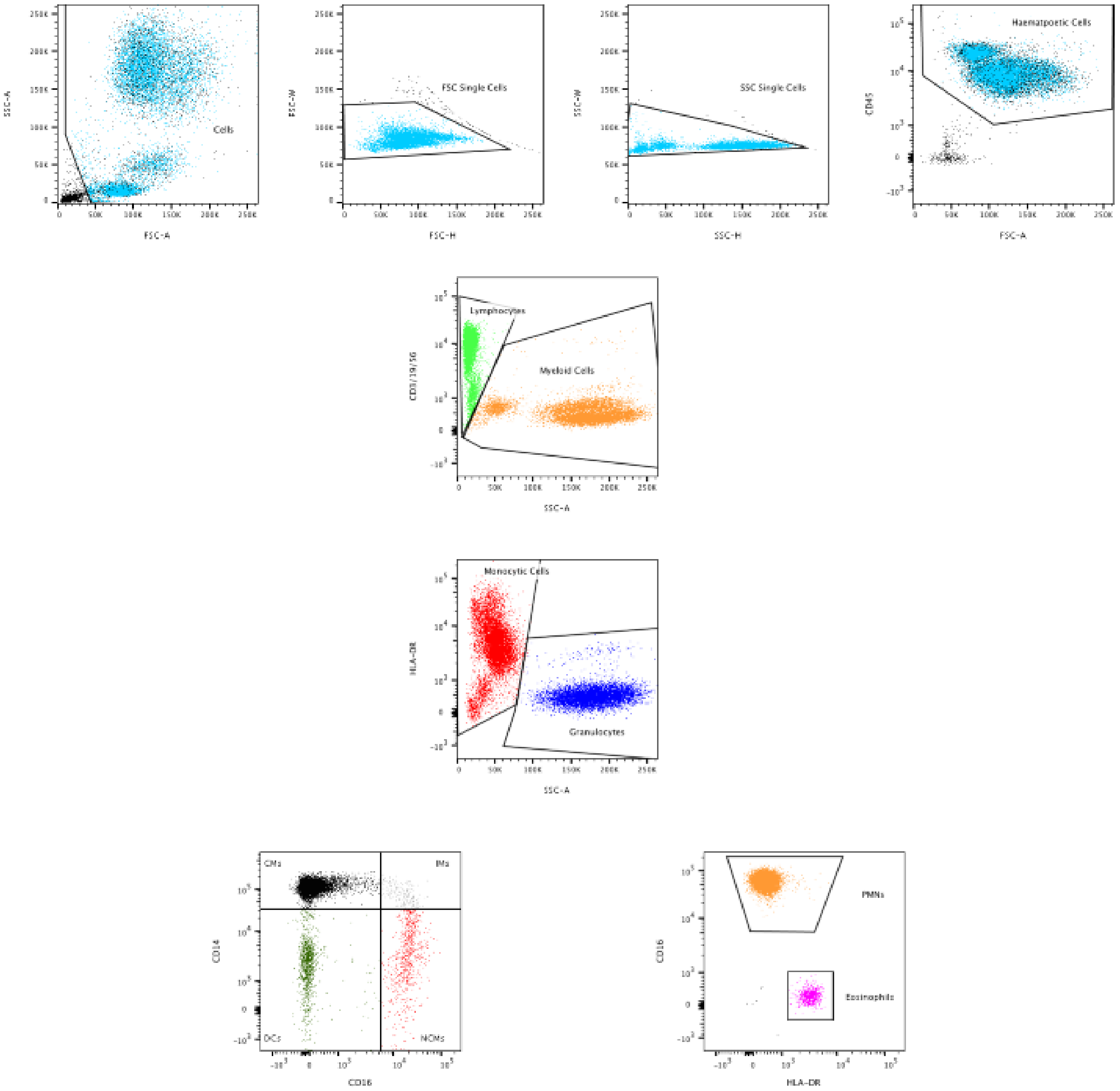

Fig. S1. Gating Strategy for Flow Cytometry of Human Peripheral Blood (Lineage Panel). Representative example of flow cytometry gating strategy for Lineage Panel (FSC, SSC, CD45-AF700, CD3-FITC, CD19-FITC, CD56-FITC, HLA-DR-BV421, CD14-BV605, CD16-APC). Exclusion of debris followed by exclusion of doublets, CD45 positive cells, lymphocyte marker (CD3, CD19, CD56) negative cells, HLA-DR status of cells, HLA-DR negative cells split by CD16 levels into PMNs (orange) (DRloCD16Hi) and Eosinophils (pink) (DRloCD16Inter), HLA-DR positive cells split by CD14 and CD16 into Classical Monocytes (CMs, black) (CD14HiCD16Lo), Non-

Classical Monocytes (NCMs, red) (CD14LoCD16Hi), Intermediate Monocytes (IMs, grey) (CD14HiCD16Hi) and Dendritic Dells (DCs, green) (CD14LoCD16Lo). Sample shown using human ACK-lysed peripheral whole blood.

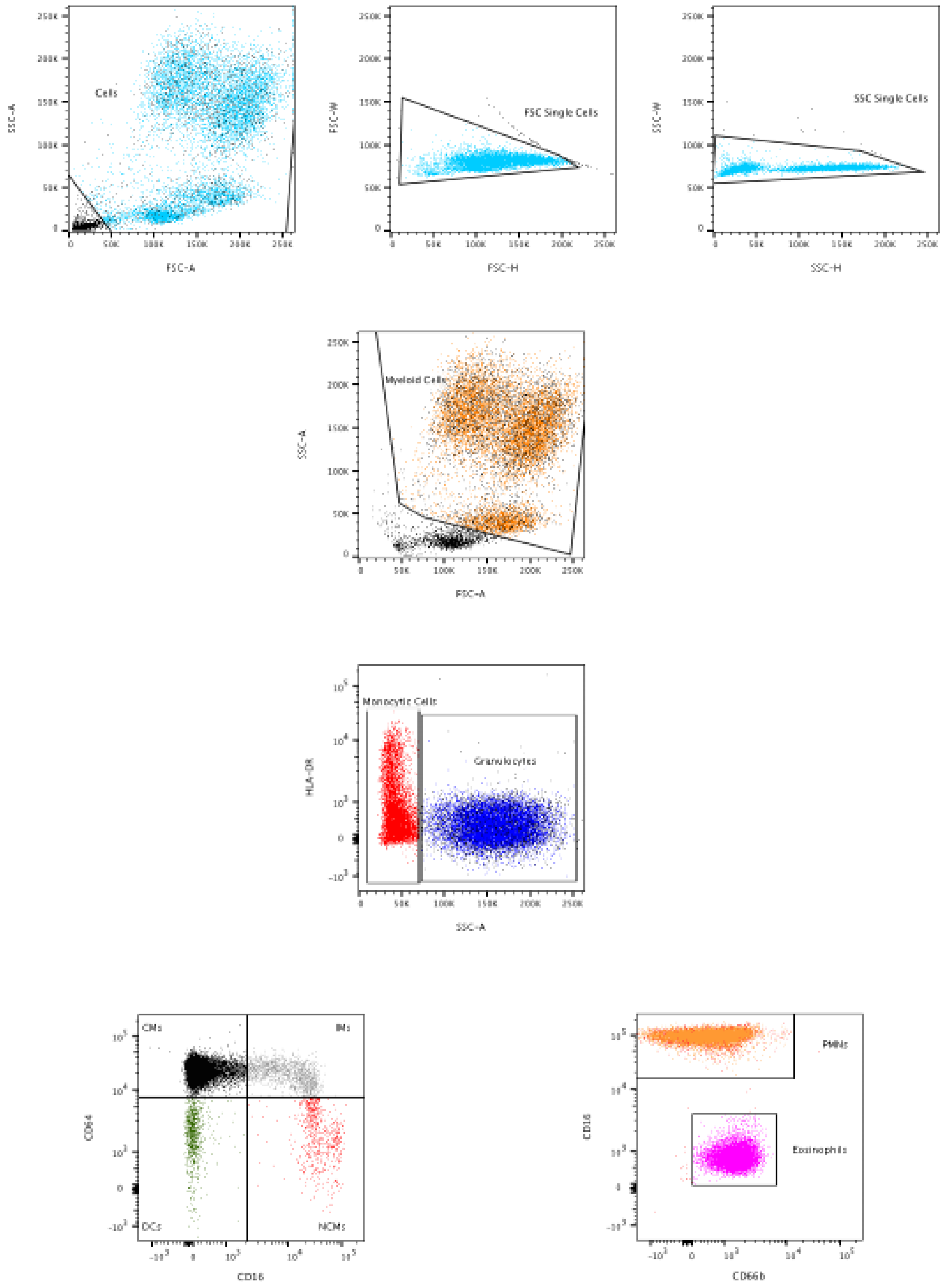

Fig. S2. Gating Strategy for Flow Cytometry of Human Peripheral Blood (Activation Panel). Representative example of flow cytometry gating strategy for Activation Panel (FSC, SSC, CD16-APC, CD11b-PerCPCy5.5, CD88-PECy7, CD54-

FITC, CD62L-BV421, CD32-PE, CD64-BV605, CDbb6-AF700, HLA-DR-APC-H7). Exclusion of debris followed by exclusion of doublets. Exclusion of lymphocytes on FSC/SSC. Division of remaining cells by SSC and CD16 into neutrophils (blue) (SSCinter-hiCD16hi) and mononuclear cells (red) (SSCloCD16lo-hi). Mononuclear cells were then further subdivided by CD64 into Classical Monocytes (CMs, black) (CD64HiCD16Lo), Non-Classical Monocytes (NCMs, red) (CD64LoCD16Hi), Intermediate Monocytes (IMs, grey) (CD64HiCD16Hi) and Dendritic Dells (DCs, green) (CD64LoCD16Lo). Sample shown using human healthy volunteer whole blood.

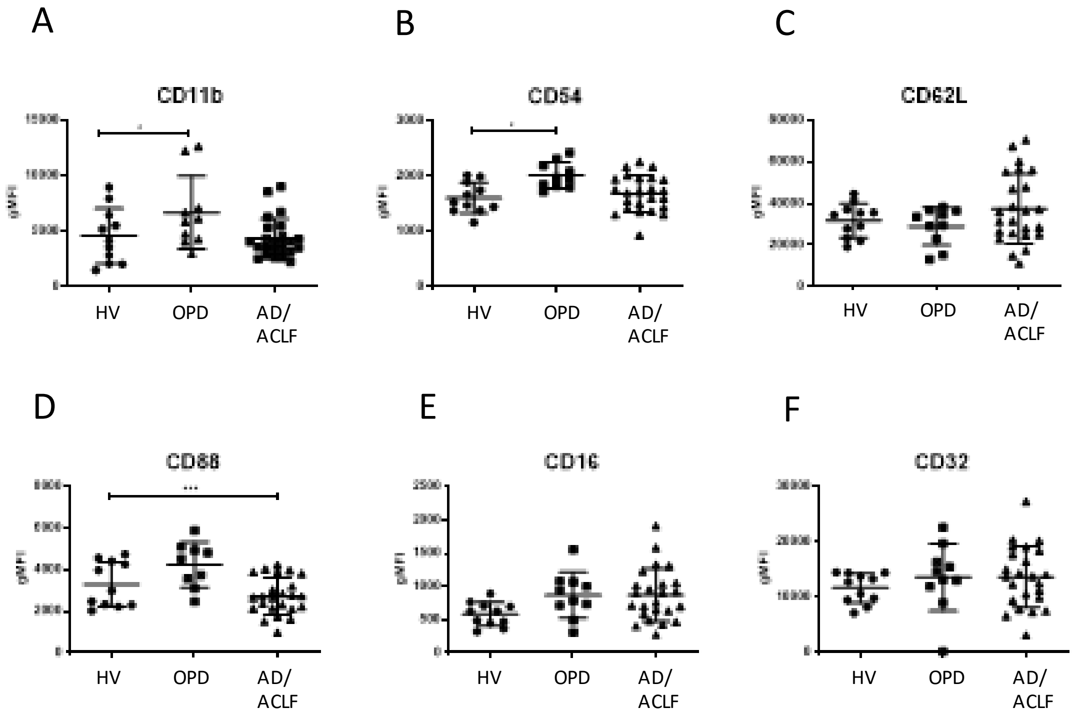

Fig. S3. (A-F) Monocytic Cell Surface Markers of Activation (labelled) with geometric Mean Fluorescence Intensity (MFI) of monocytic cells gated as Figure 7 for HV (circles), OPDs (squares), AD (triangles). Mean ± SD shown. One-way ANOVA with Tukey multiple comparisons test (all groups compared). * p<0.05, ***p<0.001.

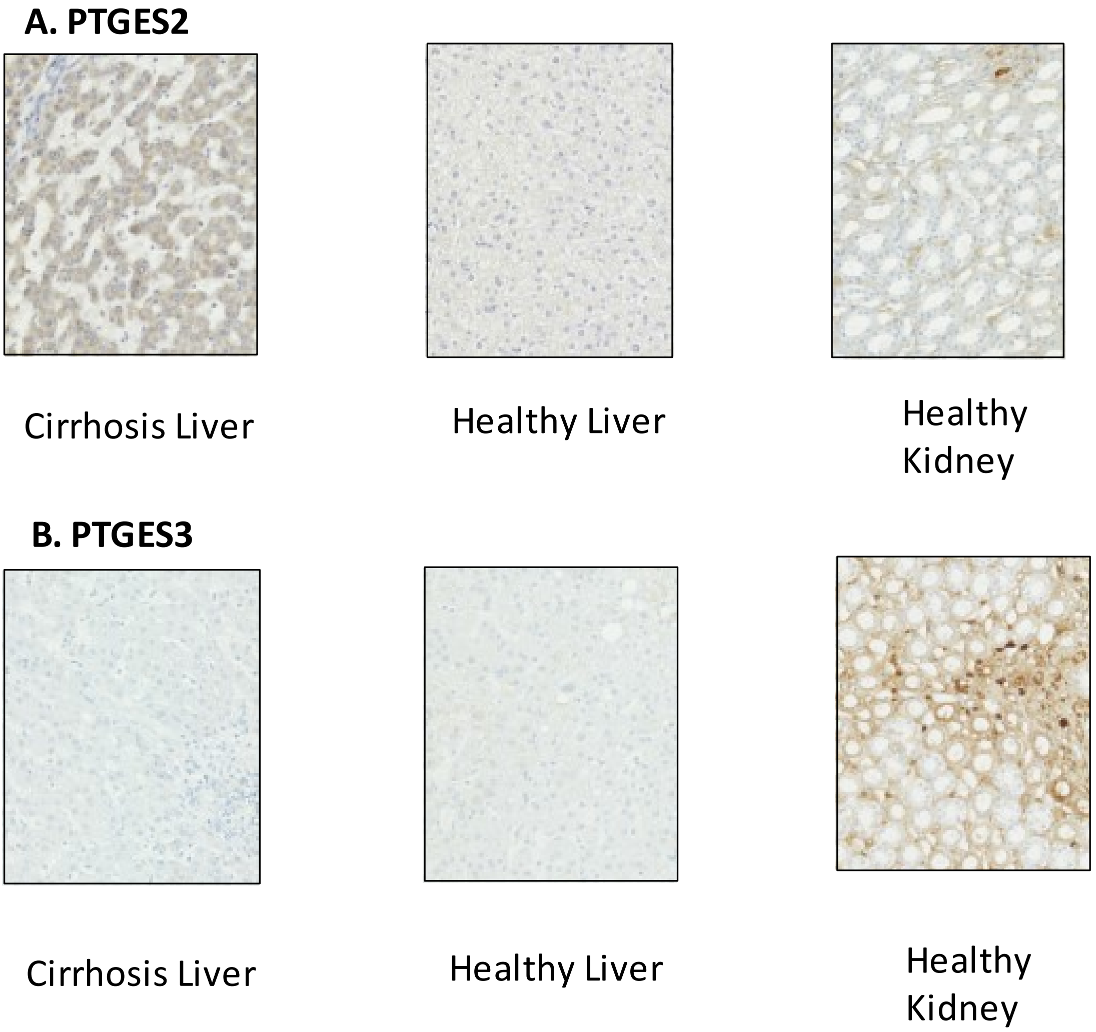

Fig. S4. Liver and Kidney immunohistochemistry Photos. (A) PTGES2 and **(B)** PTGES3 in healthy liver and kidney (positive control) and cirrhotic liver.

A. PGE2 Standard

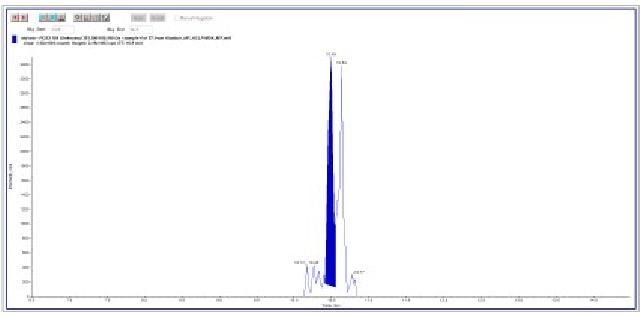

B. PGE2 in plasma from healthy volunteers

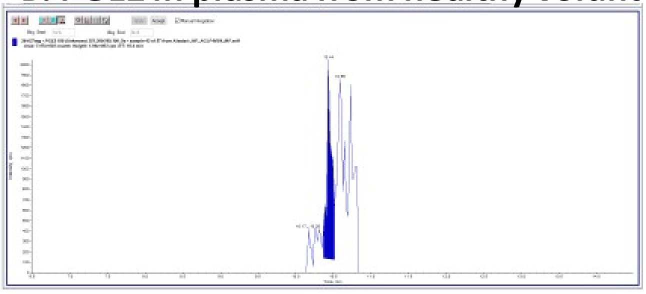

C. PGE2 in plasma from OPD

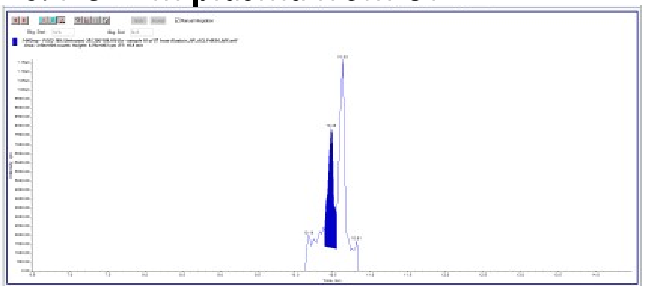

D. PGE2 in plasma from AD/ACLF

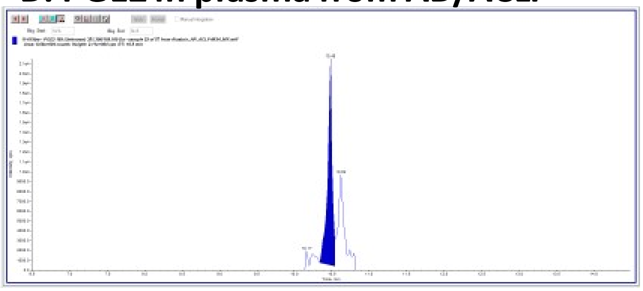

Fig. S5. Plasma PGE2 Liquid Chromatography-tandem mass spectrometry (LC-MS/MS) traces. (A) Representative Chromatogram for PGE2 Standard **(B)** Representative PGE_2 chromatogram in plasma from healthy volunteers **(C)** Representative PGE_2 chromatogram in plasma from stable live outpatients **(D)**

Representative PGE_2 chromatogram in plasma from acute decompensated liver cirrhotic patients

Supplementary tables

Table S1 A. Biochemistry data from OPD and AD/ACLF. Data displayed as medians + Interquartile range (IQR). $^{*}p<0.05$ and $^{**}p<0.01$ when OPDs compared with AD. 2 patients were receiving haemofiltration and 4 had bilirubin >150 umol/l making normal laboratory creatinine uninterpretable.

	**OPD**	**AD/ACLF**
Creatinine umol/l	107 (58-138) **	90 (56-101)**
Bilirubin umol/l	26 (16-29)	50 (24-86)
Albumin g/L	33 (32-35) *	28 (26-31)*
INR	1.2 (1.2-1.4)	1.3 (1.2-1.4)
Sodium mmol/L	138 (134-140)	137 (134-141)
C-Reactive protein	8 (3-13) *	24 (16-43)*

Table S1 B. Full blood cell count for OPD (Patients with refractory ascites attending hospital outpatient department for day case paracentesis) patients

Cohort	HB	WCC	Platelets	Neutrophils	Lymphocytes	Monocytes	Eosinophils	Basophils
OPD	68	2.36	68	1.87	0.3	0.18	0.01	0
	102	3.27	60	1.63	0.53	0.35	0.08	0.02
	80	2.95	92	1.86	0.74	0.2	0.12	0.03
	97	10.04	212	6.75	1.26	0.83	1.14	0.06
	92	4.52	111	3.43	0.45	0.55	0.05	0.04
	92	2.56	202	1.12	1.02	0.33	0.07	0.02
	89	5.31	92	4.12	0.72	0.41	0.04	0.02
	75	4.64	60	3.05	0.82	0.61	0.11	0.05
	106	5.36	130	3.92	0.52	0.71	0.17	0.04
	137	5.55	203	3.32	1.37	0.54	0.26	0.06
	115	3.52	45	1.75	1.16	0.41	0.17	0.03

Table S1C. Full Blood Count for AD/ACLF patients.

Cohort	HB	WCC	Platelets	Neutrophils	Lymphocytes	Monocytes	Eosinophils	Basophils
AD/ACLF	90	8.31	334	5.33	1.85	0.9	0.15	0.08
	109	12.66	86	10.69	0.97	0.92	0.08	0
	79	8.69	28	6.8	1.15	0.7	0.03	0.02
	90	4.49	34	3.41	0.44	0.48	0.14	0.02
	115	14.03	134	9.85	1.81	2.31	0.04	0.01
	119	16.81	207	12.89	1.83	1.93	0.07	0.08
	91	6.04	134	4.59	0.81	0.61	0.01	0.02
	64	16.96	43	14.47	1.31	1.13	0.02	0.03
	80	4.16	77	2.62	0.9	0.48	0.15	0.01
	81	7.73	117	5.89	0.84	0.71	0.28	0.01
	90	13.06	76	10.84	0.86	1.36	0	0
	81	6.19	89	3.72	1.64	0.67	0.09	0.07
	114	18.63	56	16.1	1.02	1.4	0.07	0.04
	115	13.98	335	10.58	1.61	1.61	0.17	0.01
	108	16.96	305	11.89	3.02	1.56	0.14	0.36
	106	9.7	178	7.45	1.26	0.62	0.33	0.04
	92	7.58	137	4.89	1.71	0.8	0.12	0.05
	78	7.45	120	4.98	1.08	1.06	0.3	0.03
	85	1.77	63	1.17	0.25	0.28	0.05	0.02
	81	13.01	210	9.86	1.57	1.11	0.43	0.04
	93	7.16	167	5.53	0.98	0.42	0.18	0.05
	78	3.96	118	2.33	0.67	0.63	0.29	0.04
	133	6.19	69	3.8	1.48	0.65	0.21	0.05
	137	10.15	158	6.54	1.06	2.35	0.15	0.05
	103	25	206	16.95	5.06	2.65	0.2	0.18
	91	8.69	226	6.57	1.15	0.95	0.02	0.01
	99	7.67	249	5.13	1.66	0.67	0.16	0.05
	82	8.5	86	7.14	0.52	0.72	0.05	0.06

Table S2. Antibodies and conditions for immunohistochemistry of liver and kidney tissue. Antibody targets, dilutions, source, incubation times and secondary times for immunohistochemistry of cirrhotic and healthy liver sections.

Antibody	Dilution	Source	Pretreatment	Primary Antibody incubation	Swine anti Rabbit DakoE0353
EP1 Receptor	1:100	Abcam	RCC 45 min	1h	32 min
EP2 Receptor	1:100	Abcam	RCC 45 min	1h	32 min
EP3 Receptor	1:100	Cayman	RCC 45 min	1h	32 min
EP4 Receptor	1:1000	Abcam	RCC 45 min	1h	32 min
Microsomal Prostaglandin E2 Synthase 1	1:100	Abcam	RCC 45 min	1h	32 min
Microsomal Prostaglandin E2 Synthase 2	1:100	Cayman	RCC 45 min	1h	32 min
Cytosolic Prostaglandin E2 Synthase 1	1:100	Abcam	RCC 45 min	1h	32 min

Table S3. List of primers for qPCR gene targets. Details of primers for gene targets of reference and PGE_2 pathway genes alongside amplicon length, gene ID, if primer spans exons and chromosomal location of sequence. Based on PGE_2 synthesis pathway, a number of mRNA targets were established for comparison between cohorts. 3 reference genes were chosen according to stability of expression in immune cells and Minimum Information for Publication of qPCR Experiments Guidelines. Primers were selected from ThermoFisher Scientific Taqman Database according to a number of parameters to ensure optimal qPCR quality: exon spanning probes, short amplicon length (<120 base pairs) and inventory availability.

Gene Name	Gene	Amplicon Length	UniGene ID	Exon Spanning	Chromosome Location
**Reference Genes**					
RNA polymerase III subunit A	POL3RA	89	Hs00389345_m1	Yes	Chr.10: 77975149 - 78029540
Glyceraldehyde-3-phosphate Dehydrogenase	GAPDH	74	Hs00266705_g1	Yes	Chr.12: 6534405 - 6538375
Ribosomal Protein Lateral Stalk Subunit P0	RPLP0	104	Hs99999902_m1	Yes	Chr.12: 120196700 - 120201211
**Gene Targets**					
Phospholipase A2 group Iva (PLA2)	PLA2G4 A	81	Hs00996912_m1	Yes	Chr.1: 186828900 - 186989896
Cyclooxygenase 1 (COX1)	PTGS1	60	Hs00377726_m1	Yes	Chr.9: 122369906 - 122395703
Cyclooxygenase 1 (COX2)	PTGS2	75	Hs00153133_m1	Yes	Chr.1: 186671812 - 186680427
Microsomal Prostaglandin E Synthase 1 (mPGES1)	PTGES	68	Hs00610420_m1	Yes	Chr.9: 129738336 - 129777579
Microsomal Prostaglandin E Synthase 2 (mPGES2)	PTGES2	66	Hs00228159_m1	Yes	Chr.9: 128120693 - 128128462
Cytosolic Prostaglandin E Synthase (cPGES)	PTGES3	128	Hs00832847_gH	No	Chr.12: 56663341 - 56689250
Prostaglandin E Receptor 1 (EP1)	PTGER1	85	Hs00909194_g1	Yes	Chr.19: 14472466 - 14475362
Prostaglandin E Receptor 2 (EP2)	PTGER2	79	Hs00168754_m1	Yes	Chr.14: 52314298 - 52328606
Prostaglandin E Receptor 3 (EP3)	PTGER3	131	Hs00168755_m1	Yes	Chr.1: 70852353 - 71047814
Prostaglandin E Receptor 4 (EP4)	PTGER4	68	Hs00168761_m1	Yes	Chr.5: 40679930 - 40740932
15-hydroxyprostaglandin dehydrogenase	HPGD	58	Hs00960586_g1	Yes	Chr.4: 174490177 - 174522898

Supplementary references

1. Varghese F, Bukhari AB, Malhotra R, De A. IHC Profiler: An Open Source Plugin for the Quantitative Evaluation and Automated Scoring of Immunohistochemistry Images of Human Tissue Samples. PLOS ONE. 2014 May 6;9(5):e96801.

2. Fuhrich DG, Lessey BA, Savaris RF. Comparison of HSCORE Assessment of Endometrial β3 Integrin Subunit Expression with Digital HSCORE Using Computerized Image Analysis (ImageJ). Anal Quant Cytopathol Histopathol. 2013 Aug;35(4):210–6.

3. Braun M, Kirsten R, Rupp NJ, Moch H, Fend F, Wernert N, et al. Quantification of protein expression in cells and cellular subcompartments on immunohistochemical sections using a computer supported image analysis system. Histol Histopathol. 2013;28(5):605–1048, Weiss, Front Immunol 2021
