# Supplementary material for: Monocyte dysfunction in decompensated cirrhosis is mediated by the prostaglandin E2-EP4 pathway
Source: JHEP Rep. 2021 Aug 4;3(6):100332. doi: 10.1016/j.jhepr.2021.100332 (PMC8603213; doi:10.1016/j.jhepr.2021.100332)
Supplement: Multimedia component 2 [file mmc2.pdf]

JOURNAL
OF HEPATOLOGY

Journal of Hepatology

CTAT methods

Tables for a "Complete, Transparent, Accurate and Timely account" (CTAT) are now mandatory for all revised submissions. The aim is to enhance the reproducibility of methods.

- Only include the parts relevant to your study
- Refer to the CTAT in the main text as 'Supplementary CTAT Table'
- Do not add subheadings
- Add as many rows as needed to include all information
- Only include one item per row

If the CTAT form is not relevant to your study, please outline the reasons why:

1.1 Antibodies

Name	Citation	Supplier	Cat no.	Clone no.
EP1 Receptor		Abcam		
EP2 Receptor		Abcam		
EP3 Receptor		Cayman		
EP4 Receptor		Abcam		
Microsomal Prostaglandin E2 Synthase 1		Abcam		
Microsomal Prostaglandin E2 Synthase 2		Cayman		
Cytosolic Prostaglandin E2 Synthase 1		Abcam		
EP1 Receptor		Abcam		

1.2 Cell lines

Name	Citation	Supplier	Cat no.	Passage no.	Authentication test method

1.3 Organisms

Name	Citation	Supplier	Strain	Sex	Age	Overall n number

1.4 Sequence based reagents

Name	Sequence	Supplier

1.5 Biological samples

Description	Source	Identifier

1.6 Deposited data

Name of repository	Identifier	Link

1.7 Software

Software name	Manufacturer	Version

1.8 Other (e.g. drugs, proteins, vectors etc.)

1.9 Please provide the details of the corresponding methods author for the manuscript:

Name:
Alastair O'Brien
Address:
UCL Institute for Liver and Digestive Health
Royal Free Campus
Rowland Hill Street
London NW3 2PF
Telephone:
+44 7799413861
Email:
a.o'brien@ucl.ac.uk

2.0 Please confirm for randomised controlled trials all versions of the clinical protocol are included in the submission. These will be published online as supplementary information.

